# GMStool: GWAS-based marker selection tool for genomic prediction from genomic data

**DOI:** 10.1038/s41598-020-76759-y

**Published:** 2020-11-12

**Authors:** Seongmun Jeong, Jae-Yoon Kim, Namshin Kim

**Affiliations:** 1 Genome Editing Research Center, Korea Research Institute of Bioscience and Biotechnology (KRIBB), Daejeon, 34141 Republic of Korea; 2 Department of Bioinformatics, KRIBB School of Bioscience, University of Science and Technology (UST), Daejeon, 34141 Republic of Korea

**Keywords:** Computational biology and bioinformatics, Computational models, Data mining, Software, Bioinformatics

## Abstract

The increased accessibility to genomic data in recent years has laid the foundation for studies to predict various phenotypes of organisms based on the genome. Genomic prediction collectively refers to these studies, and it estimates an individual’s phenotypes mainly using single nucleotide polymorphism markers. Typically, the accuracy of these genomic prediction studies is highly dependent on the markers used; however, in practice, choosing optimal markers with high accuracy for the phenotype to be used is a challenging task. Therefore, we present a new tool called GMStool for selecting optimal marker sets and predicting quantitative phenotypes. The GMStool is based on a genome-wide association study (GWAS) and heuristically searches for optimal markers using statistical and machine-learning methods. The GMStool performs the genomic prediction using statistical and machine/deep-learning models and presents the best prediction model with the optimal marker-set. For the evaluation, the GMStool was tested on real datasets with four phenotypes. The prediction results showed higher performance than using the entire markers or the GWAS-top markers, which have been used frequently in prediction studies. Although the GMStool has several limitations, it is expected to contribute to various studies for predicting quantitative phenotypes. The GMStool written in R is available at www.github.com/JaeYoonKim72/GMStool.

## Introduction

Genomic prediction (GP) based on single nucleotide polymorphism (SNP) markers has become a powerful tool for various human healthcare as well as conventional plant and animal breeding programs^1, 2^. With the recent dramatic decreases in sequencing and genotyping costs, GP is more readily accessible than ever and has enabled the efficient prediction of genetic disease risks, genomic breeding values, and complex quantitative phenotypes directly from genomic data. GP can be performed using either all SNPs or only subset SNPs from whole-genome sequencing (WGS) or SNP array data. Generally, since many SNPs are used, using all SNPs from WGS data is expected to result in higher GP accuracy than using SNPs from array data. However, in practice, this increase in accuracy is rarely observed in real data^3^, and little differences, or even a decrease in accuracy, have been reported in previous studies^4, 5^. This is because WGS data retains more SNPs relating to a phenotype of interest, but the number of relevant SNPs accounts for a relatively small proportion of the total number of SNPs used, and the considerable number of remaining SNPs are phenotypically neutral^6^. To avoid these burdens due to the large number of uninformative SNPs, approaches using an adequate subset of SNPs have been proposed^6^, and multiple studies have reported that SNP subsets improved GP accuracies compared to using all or numerous SNPs. For example, Ni et al*.* improved GP accuracy using only SNPs in or around genes from WGS data^7^, and Brondum et al*.* increased GP accuracy using both SNPs associated with quantitative trait loci and SNPs significantly detected in a genome-wide association study (GWAS)^8^.

A GWAS identifies SNP markers associated with a phenotype. Thus, it is utilized as a useful approach to construct a subset of SNP markers for GP. Usually, significant SNPs detected below the *p*-values of 1** × **10^–6^ or 5** × **10^–8^ are used as a subset, and simulation studies have reported that improved accuracy is attained when using GWAS-significant SNPs and their surrounding SNPs^9^. However, these predictions using the GWAS-significant and/or surrounding SNPs have not always been successful^10^. This is likely because the significant SNPs explain only a small percentage of the total genetic variation for a phenotype^11^, and each SNP also accounts for a small portion of the phenotypic variance^12^. Thus, identifying more robust methods to select the optimal subset of SNPs for GP has focused on multiple studies. Bermingham et al*.* and Filho et al*.* reported that using the top 100–10,000 GWAS SNP markers as subsets could increase GP accuracy compared to using only GWAS-significant SNPs^13, 14^. Yilmaz presented an algorithm that selects subsets of markers considering SNP–SNP interactions from a GWAS result and confirmed that using these subsets improves accuracy compared to using only top GWAS SNPs^15^. These studies have demonstrated that the selection of SNP subset has a considerable effect on GP accuracy; however, the GWAS top SNP-based approaches have difficulty selecting an appropriate number of top SNPs with high GP accuracy and require numerous attempts each time for each phenotype. The interaction-based GWAS approach requires information on the interactions between SNPs, in addition to the GWAS result. Furthermore, if the interaction information is incomplete, this approach can construct a biased subset that can hinder GP accuracy^15^. Therefore, there is a need for a new approach that selects an optimal subset of SNPs to maximize GP accuracy while considering the single and interaction effects of markers.

In this study, we have developed an implemented GWAS-based marker selection tool named GMStool. GMStool searches for SNP markers in order of the lowest *p*-value in the GWAS result and constructs the optimal marker set by accumulating SNP markers that increase the phenotype's prediction accuracy. Subsequently, GMStool performs GP modeling and presents the best prediction model with the optimal marker set. Statistical, machine, and/or deep learning methods are used, and the interaction effects of the SNP markers are considered indirectly through modeling. The R package and execute scripts of GMStool are available at www.github.com/JaeYoonKim72/GMStool with detailed usage instructions.

## Methods

### GMStool scheme: preparation

**Figure 1:**
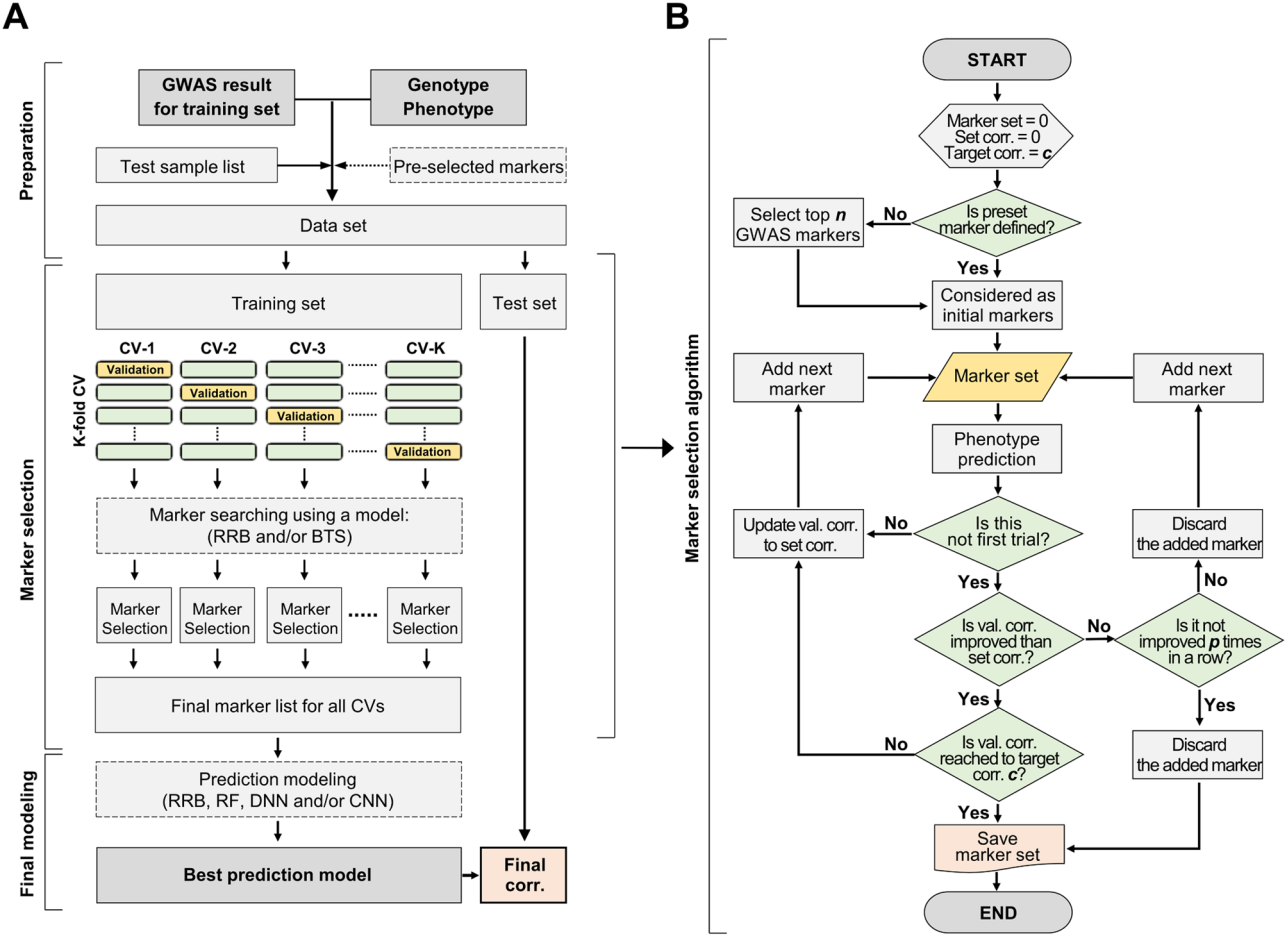
Scheme of GMStool. (**A**) Overall workflow. GMStool consists of three phases: preparation, marker selection, and final modeling. The dotted rectangles indicate the options that users can choose: whether to define pre-selected markers and which model to use for the marker selection and final modeling phases. (**B**) A brief algorithm for the marker selection phase. "preset marker” in the first conditional statement means the "pre-selected markers” in the overall workflow. Abbreviations ‘Set. corr.’ ‘Target corr.’, and ‘val. corr.’ mean ‘correlation rate of marker set’, ‘target correlation rate’, and ‘correlation rate of validation set’, respectively.

GMStool consists of three phases: preparation, marker selection, and final modeling (Fig. 1A). In the preparation phase, GMStool requires four inputs: genotype, phenotype, GWAS result, and test sample list files. The genotype file consists of markers (rows) and samples (columns), and genotypes are coded as − 1, 0, 1, and 2 for missing, homozygous reference, heterozygous, and homozygous alternative genotypes, respectively. The phenotype file is similar to the genotype file, but its rows and columns consist of samples and phenotypes. The GWAS result file consists of four columns: marker name, chromosome number, physical position, and *p*-value columns. The test list file consists of a single column with the names of the test samples. As an option, a list of markers that must be included in the final modeling can be passed to GMStool. If this file named by 'preset marker’ is not defined, the top *n* markers based on GWAS *p*-values are selected and used for the initial analysis (default 1). Note that GWAS must be performed using only a training set (excluding the test set) to completely exclude information on the test set that may cause bias in the training result^16^. Although the GWAS does not include the test set, both genotype and phenotype files must include all samples of the training and test set, since GMStool automatically converted the test set into input files for the final modeling phase.

### GMStool scheme: marker selection

The marker selection phase applies the forward selection method of regression analysis and sequentially selects SNP markers that increase the correlation rate between observed and predicted phenotypes on the validation set (Fig. 1B). The optimal marker set for the final modeling phase is gradually constructed by accumulating SNPs one by one. The metric is used as the Pearson's correlation rate instead of accuracy due to the continuous phenotypes. Depending on the forward selection method, SNPs that have not been selected once are not selected again in the selection process. The ridge regression best linear unbiased prediction (RRB) and bootstrap trees (BTS) methods are provided as learning models, and either one or both models can be used. If both methods are selected, BTS is calculated sequentially after RRB, and the union of the SNP markers derived from the two methods is presented as an optimal marker set. The overall workflow of the marker selection phase is as follows (Fig. 1A):

1. Divide the input data into training and test sets, using the information of the test set defined by the user.
2. Divide the training set into *k* groups for cross-validation (CV; default 5), and perform marker selection in each group. All *k* groups are executed simultaneously through multi-threading. The process of selecting markers in each group is as follows (Fig. 1B):
  A. Consider one sub-group as a validation set and the remaining sub-groups as a training set.
  B. Build the prediction model using all the markers in the training set, according to the user's selection model. Then, calculate the correlation rates on the training and validation sets, respectively (optional).
  C. Select the top *n* initial markers from GWAS results ordered by *p*-values (default 1), and consider them as an initial marker set. If ‘preset marker’ is defined, consider these preset markers as the initial marker set instead of selecting the top markers. Build the prediction model using the initial markers of train sets and predict the correlation rate on the validation set. Place the initial markers into the selected marker set (yellow parallelogram in Fig. 1B; ‘Marker set’) and the initial correlation rate into the correlation rate for the selected marker set (gray hexagon in Fig. 1B; ‘Set corr.’).
  D. If the top *n* initial markers were selected, select the next marker (*n* + 1), and if preset markers were defined, select the top one from GWAS, ordered by *p*-values. Add this marker to the selected marker set formed in step C. Build the prediction model using the marker set, and calculate the current correlation rate on the validation set. If the difference of the correlation rate between ‘current’ and ‘previous (Set corr.)’ is greater than or equal to the increment value $\updelta$ (default 0.00005), place the marker into the selected marker set (‘Marker set') and update the previous correlation rate to the current correlation rate ('Set corr'); otherwise, the marker is discarded from the selected marker set, and the current correlation rate is ignored.
  E. Iterate step D, while adding markers one by one. If the correlation rate of the selected marker set on the validation set reaches the target correlation rate defined by the user (‘Target corr’; default 1.0), stop marker selection and return the final marker list. As a stop condition, if the validation set's correlation rates do not improve *p* times in a row, that is, if the markers are not selected *p* times in succession, stop the marker selection and return the results up to that point. Here, *p* is a number corresponding to *x*% of the total number of input markers and is automatically calculated according to the *x* defined by the user (default 20).
3. Integrate the selected marker lists of *k* groups into one final marker list of all *k* CVs. Using both RRB and BTS methods, one final marker list is generated by combining all marker lists derived from these two methods. Subsequently, the input files for the final modeling phase are made based on the final marker list.

### GMStool scheme: final modeling

The final modeling phase performs prediction modeling using RRB, random forest (RF), deep neural network (DNN), and convolution neural network (CNN) models (Fig. 1A). All four models, or a subset of them, can be used. Prediction modeling for each model is conducted by repeating *p* times (default 50), and in each *p*, training and validation sets are randomly constructed at a ratio of 8:2 from the training set derived from the marker selection phase. Of all the *p* modeling iterations in each model, the model with the highest correlation rate on the validation set is considered the final prediction model. After modeling, a final prediction is performed on the test set, and its correlation rate between the observed and predicted phenotypes is presented with the saved model file and summary plots. If more than one model is selected, the model with the highest correlation rate on the validation set is presented as the best model.

### Models of GMStool: RRB

RRB, a statistical model, assumes that marker effects follow a normal distribution with constant variance and all effect sizes are small and similar. This model is implemented using the “rrBLUP” library^17^ in R^18^, and is used in GMStool as follows: $\mathbf{y}={\varvec{\mu}}+\mathbf{X}{\varvec{\beta}}+\mathbf{e},$ where $\mathbf{y}$ is an $n\times 1$ vector of phenotype values (*n*; samples), ${\varvec{\mu}}$ is an $n\times 1$ mean vector of phenotype values, $\mathbf{X}$ is an $n\times p$ matrix of genotype markers (*p*; markers), ${\varvec{\beta}}$ is an *n* $\times 1$ vector of marker effects, and $\mathbf{e}$ is an *n* $\times 1$ vector of error effects with $\mathbf{e}\sim \boldsymbol{\rm N}(0,\boldsymbol{ }\boldsymbol{\rm I}{{\varvec{\sigma}}}_{{\varvec{e}}}^{2}$**)**. Here, the loss function is $\mathcal{L}\left({\varvec{\beta}}\right)={\left({\varvec{y}}-{\varvec{X}}{\varvec{\beta}}\right)}^{T}\left({\varvec{y}}-{\varvec{X}}{\varvec{\beta}}\right)+\lambda {{\varvec{\beta}}}^{T}{\varvec{\beta}},$ and ${\varvec{\beta}}$ is derived as $\widehat{{\varvec{\beta}}}={\left({{\varvec{X}}}^{T}{\varvec{X}}+\lambda {\varvec{I}}\right)}^{-1}{{\varvec{X}}}^{T}{\varvec{y}}$. The penalty parameter $\lambda$ is estimated from the training data under the RRB model^19^. The ${\varvec{\beta}}$ values estimated from the training set are used to predict phenotype values for the validation and test datasets.

### Models of GMStool: RF and BTS

RF, a decision tree-based machine learning model, provides an explicit representation of markers' interactions without needing to pre-define the interactions. Thus, RF can reflect SNP-SNP interactions when modeling genotype data^20^. The RF for regression trees in GMStool is implemented through the “randomForest”^21^ library in R^18^ and used in the final modeling phases as follows:

1. Generate 1000 bootstrap datasets by sampling the training samples with a 0.632 ratio.
2. Construct regression trees by sampling 1/3 of the total input markers for each dataset.
3. Grow the regression trees by splitting markers, with the loss function of the root mean square error.
4. Derive the predicted phenotypes of the validation and test sets by averaging the phenotype values predicted from the 1000 trees.

In the RF model, it is possible that none of the specific markers will be included in every tree due to marker sampling. Therefore, the marker selection phase uses the BTS model, an RF model that excludes the marker sampling function, and only the sample bootstrap function remains. The BTS model uses 100 trees sampled at a 0.632 ratio from the training samples and grows the trees without marker sampling. Then, in the same way as RF, the BTS model calculates the validation set's predicted phenotypes.

### Models of GMStool: DNN and CNN

**Figure 2:**
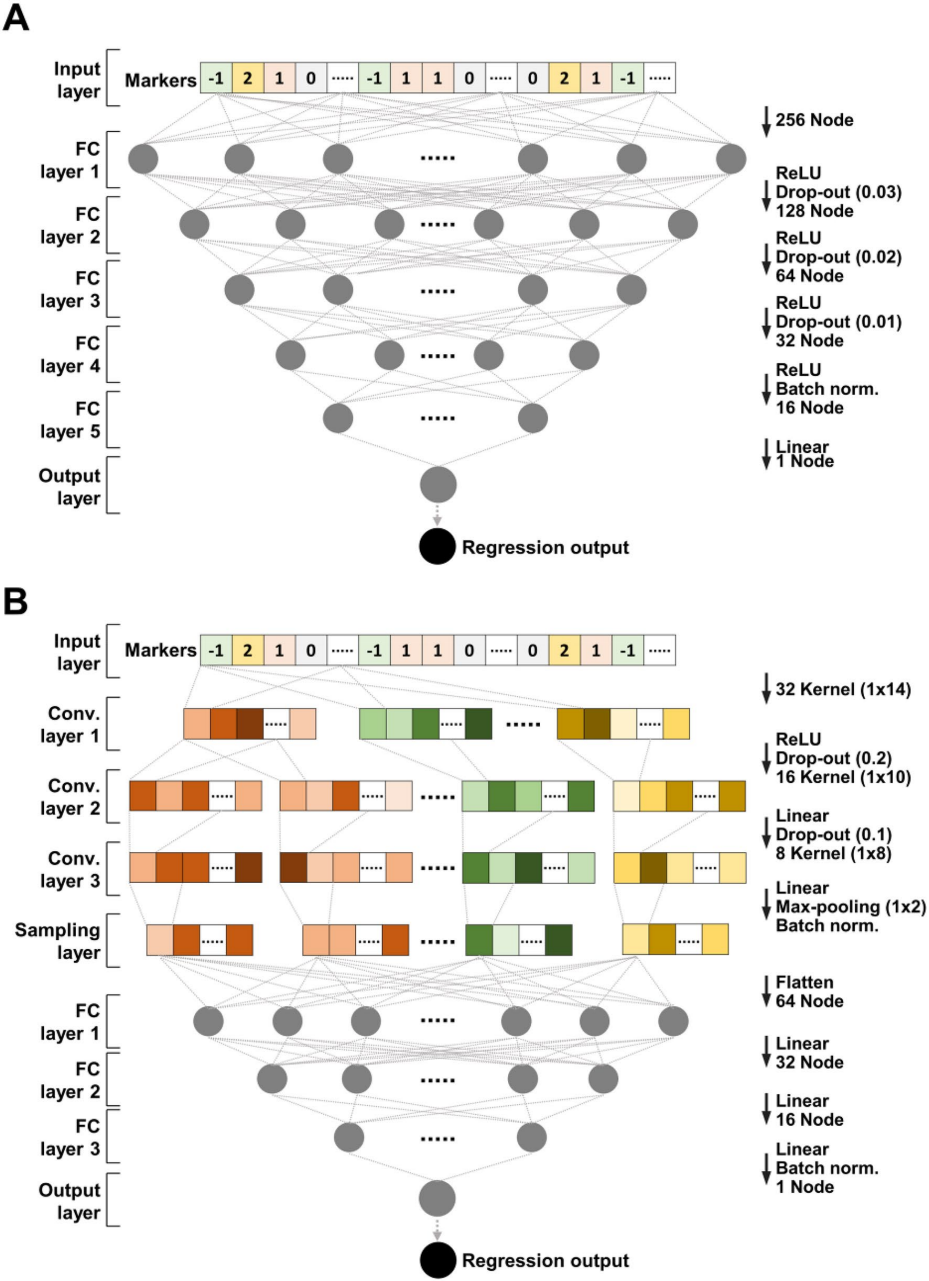
Architectures of the DNN and CNN regression models for the final modeling phase. (**A**) DNN model has five fully connected layers, and (**B**) CNN model has three convolution layers and three fully connected layers. The drop-out rates and kernel sizes are indicated in parentheses at the right of the figures. *FC *full connection and *Conv* convolution, respectively.

DNN and CNN, two deep learning models, are implemented using the "tensorflow”^22^ and "keras”^23^ libraries in R^18^. These two models can take SNP-SNP interactions into account during modeling and computed at high speed through a graphics processing unit (GPU)^24^. The DNN model in GMStool has a 256-128-64-32-16-1 architecture: one input layer with the number of neurons equal to marker size, five fully connected layers with 256, 128, 64, 32, and 16 neurons, respectively, and one output layer with one neuron (Fig. 2A). The architecture of the CNN is constructed as a 32-16-8-64-32-16-1: one input layer with the number of neurons equal to marker size, three convolution layers with 32, 16, and 8 kernels, one sampling layer, three fully connected layers with 64, 32, and 16 neurons, respectively, and one output layer with one neuron (Fig. 2B). To prevent overfitting, dropout layers are applied to the DNN and CNN models. Parameters are optimized using the AdaMax algorithm^25^ with a learning rate of 0.001 on DNN and 0.003 on CNN, through a maximum of 1000 epochs. Batch sizes are set to one-twentieth of the input samples, and the loss function is the mean squared error. During optimization, an early stop of 30 epochs and a learning rate decay of 0.0003 are applied to reduce the computational time and avoid overfitting. In using the CNN, input markers are automatically sorted in ascending order according to chromosome and physical numbers to consider the interactions between adjacent markers effectively. The other models, including DNN, uses input markers sorted in the most selected order among all CVs. After modeling, phenotype predictions are performed on the validation and test sets.

### Evaluation data and GWAS

Rice (*Oryza sativa*) and soybean (*Glycine max*) data were used to evaluate the performance of GMStool (Table 1). The rice data for both genotypes and phenotypes were obtained from www.ricediversity.org^26^. The soybean data for genotypes were obtained from www.soybase.org/data/public/Glycine_max/Wm82.gnm2.div.L78C^27^, and its phenotype data was shared from our previous study^28^. In the present study, the rice genotype data consisted of 413 samples with 44,100 SNPs, and the soybean genotype data consisted of 1928 samples with 170,223 SNPs. SNPs with a minor allele frequency (MAF) of < 1% were filtered out, and 36,901 and 95,776 SNPs with missing rates of 0.043 and 0.005 were obtained from the rice and soybean datasets, respectively. Imputation was then conducted on both datasets using BEAGLE v5.1^29^. For the phenotype data, the days to flowering time (DTF), protein content (PC), and plant height (PH) were used for rice, and DTF was used for soybean.

**Table 1:** Samples, markers, and phenotypes for the rice and soybean datasets.

Data	Samples	Markers^a^	Phenotype	Samples	Median	Mean	Std
Rice	413	36,901/44,100	DTF	305	74.00	71.77	8.51
			PC	383	117.50	116.58	21.09
			PH	393	8.45	8.59	0.94
Soybean	1928	95,776/170,223	DTF	1,861	46.00	44.87	6.34

^a^Number in front of “/” indicates the number of markers filtered with MAF < 1%, and the number behind indicates the number of all markers.

Prior to the GWAS, the test sets of each dataset were randomly selected at a rate of 20% (Table 2). For the DTF, PC, and PH of rice, the test sample sizes were 61, 76, 78, and the training sample sizes were 244, 307, and 315, respectively. Soybean, the larger dataset, consisted of 1489 training and 372 test samples. The test set samples were representative of the genomic diversity of the total samples, as shown by their even distribution without a bias in the principal component analysis based on population structure (Supplementary Fig. S1).

**Table 2:** Selected marker sets and correlation rates for all CVs.

Data	Phenotype	Method	Train/val./test samples	Selected/all markers^a^	Train corr (mean ± std.)^b^	Val. Corr (mean ± std.)^b^
Rice	DTF	RRB	163/81/61	746/36,901	0.994 ± 0.007	0.986 ± 0.011
		BTS	163/81/61	120/36,901	0.946 ± 0.016	0.839 ± 0.047
		RRB and BTS	163/81/61	817/36,901	0.970 ± 0.028	0.913 ± 0.086
	PC	RRB	205/102/76	805/36,901	0.988 ± 0.016	0.990 ± 0.005
		BTS	205/102/76	114/36,901	0.951 ± 0.006	0.841 ± 0.024
		RRB and BTS	205/102/76	873/36,901	0.970 ± 0.023	0.912 ± 0.105
	PH	RRB	210/105/78	620/36,901	0.982 ± 0.013	0.992 ± 0.007
		BTS	210/105/78	115/36,901	0.970 ± 0.002	0.883 ± 0.035
		RRB and BTS	210/105/78	675/36,901	0.976 ± 0.010	0.938 ± 0.063
SOY	DTF	RRB	1,191/298/372	2,126/95,776	0.953 ± 0.014	0.993 ± 0.001
		BTS	1,191/298/372	224/95,776	0.922 ± 0.020	0.842 ± 0.040
		RRB and BTS	1,191/298/372	2,256/95,776	0.938 ± 0.023	0.917 ± 0.097

*val.* mean validation and *corr.* correlation rate, respectively.

^a^Selected markers were derived from the union of markers selected from all CVs.

^b^Average and standard deviation of correlation rates for all CVs.

**Figure 3:**
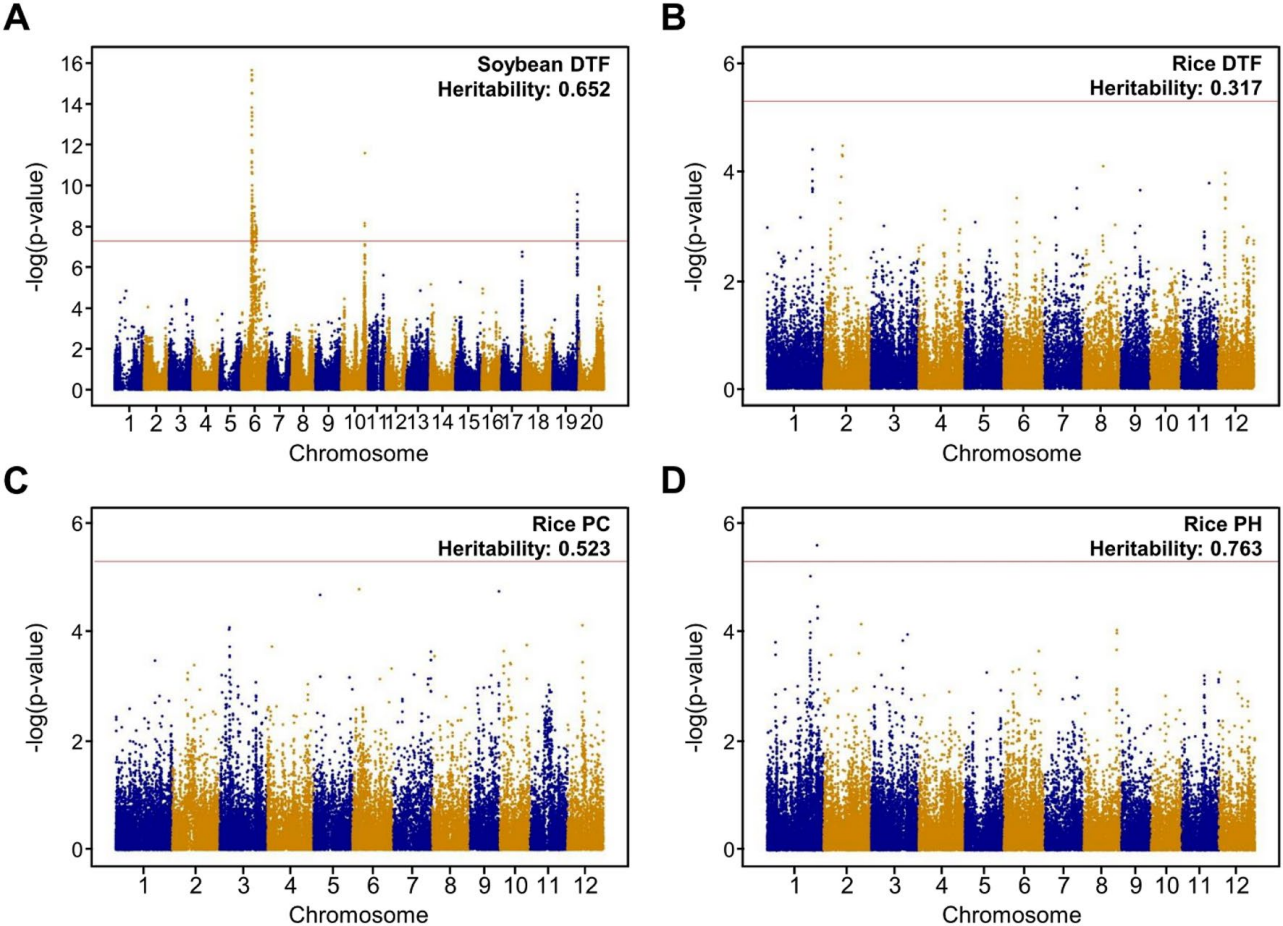
GWAS results for the soybean and rice datasets. (**A**) Manhattan plot for soybean DTF. (**B–D**) Manhattan plots for rice DTF, PC, and PH. Statistical significant cut-offs for soybean and rice data are − log(5 × 10^–8^) and − log(5 × 10^–6^), respectively. The heritabilities of phenotypes are shown at the upper right of the figures.

GWASs were conducted for each training set using a mixed linear model of GAPIT v3, which adjusts the kinship and population structures^30^ (Fig. 3 and Supplementary Table S1). Considering the number of MAF-filtered markers, statistically significant *p*-values were set at 5 × 10^–6^ and 5 × 10^–8^ in the rice and soybean datasets. All heritabilities were measured as the proportion of the total phenotypic variance explained by the genotypic variance.

## Results

### GWAS

In the rice data, PH showed one GWAS-significant SNP with a high heritability of 0.763, while DTF and PC showed no significant markers with relatively low heritabilities of 0.317 and 0.523, respectively (Fig. 3). Unlike rice-DTF, soybean-DTF had a high heritability of 0.652 and showed 105 GWAS-significant SNPs. In terms of heritability, these GWAS results confirmed that soybean-DTF and rice-PH are greatly affected by genetic effects and have the potential for GP with relatively high accuracy. Rice DTF and PC were confirmed to have low genetic effects, but they were used to compare differences in GP with heritability.

### Marker selection

Marker selection for each phenotype was performed based on the genotype, phenotype, test sample list, and GWAS result files. Considering the training sample sizes, CV values of 3 and 5 were set for the rice and soybean datasets, respectively. The training and validation sizes in each CV were 163 and 81 in rice-DTF, 205 and 102 in PC, 210 and 105 in PH, and 1,191 and 298 in soybean-DTF, respectively (Table 2). Other selection options were adopted as follows: 1.0 target correlation rate, 0.00005 increment, and one initial marker (− c, − d, and − is; 1.0, 0.00005, and 1). In addition, the stop option was applied, which terminates the selection if SNPs are not continuously selected as many as a number corresponding to 20% of the total input markers (− x; 20). All selection methods were adopted for the method option for comparisons (− m; RRB, BTS, and RRB_BTS). As a result, the number of selected markers for the three methods were 746-120-817, 805-114-873, and 620-115-675 in the rice DTF, PC, and PH phenotypes, respectively, and 2126-224-2256 in the soybean DTF phenotype (Table 2 and Supplementary Table S2). When both RRB and BTS were used, the largest number of markers were selected, and in a separate method, more markers were selected when using RRB than BTS. In the case of rice, the higher the heritability, the fewer markers tended to be selected. The correlation rates between the observed and predicted phenotypes of the validation sets ranged from a minimum of 0.839 (rice-DTF and BTS) to a maximum of 0.992 (PH and RRB). Although some of the correlation rates were not relatively high, each selection method selected as many potential markers as possible under the target correlation rate of 1.0. During the rice phenotypes selection process, the BTS method read all 36,901 input markers for all CVs, and the RRB method read all markers for 1 or 2 CVs (out of 3 CVs). The rest of the CVs were stopped according to the stop condition since their markers were not selected 7380 times in succession. In soybean DTF, this stop condition was applied to all 5 CVs of both methods because their markers were also not selected 19,155 times consecutively in all CVs. As for the average calculation time per CV, RRB took from 4 h 52 m (rice-DTF) to 15 h 6 m (soybean-DTF), and BTS took from 4 h 25 m (rice-DTF) to 8 h 2 m (soybean-DTF), using the Intel Xeon E5-2680 central processing unit (CPU) (Supplementary Table S3). Using both methods took a minimum of 9 h 24 m (rice-DTF) and a maximum of 23 h 32 m (soybean-DTF). The calculation time of RRB took longer than that of BTS, and they all tended to require more time with larger sample sizes.

### Final prediction

**Figure 4:**
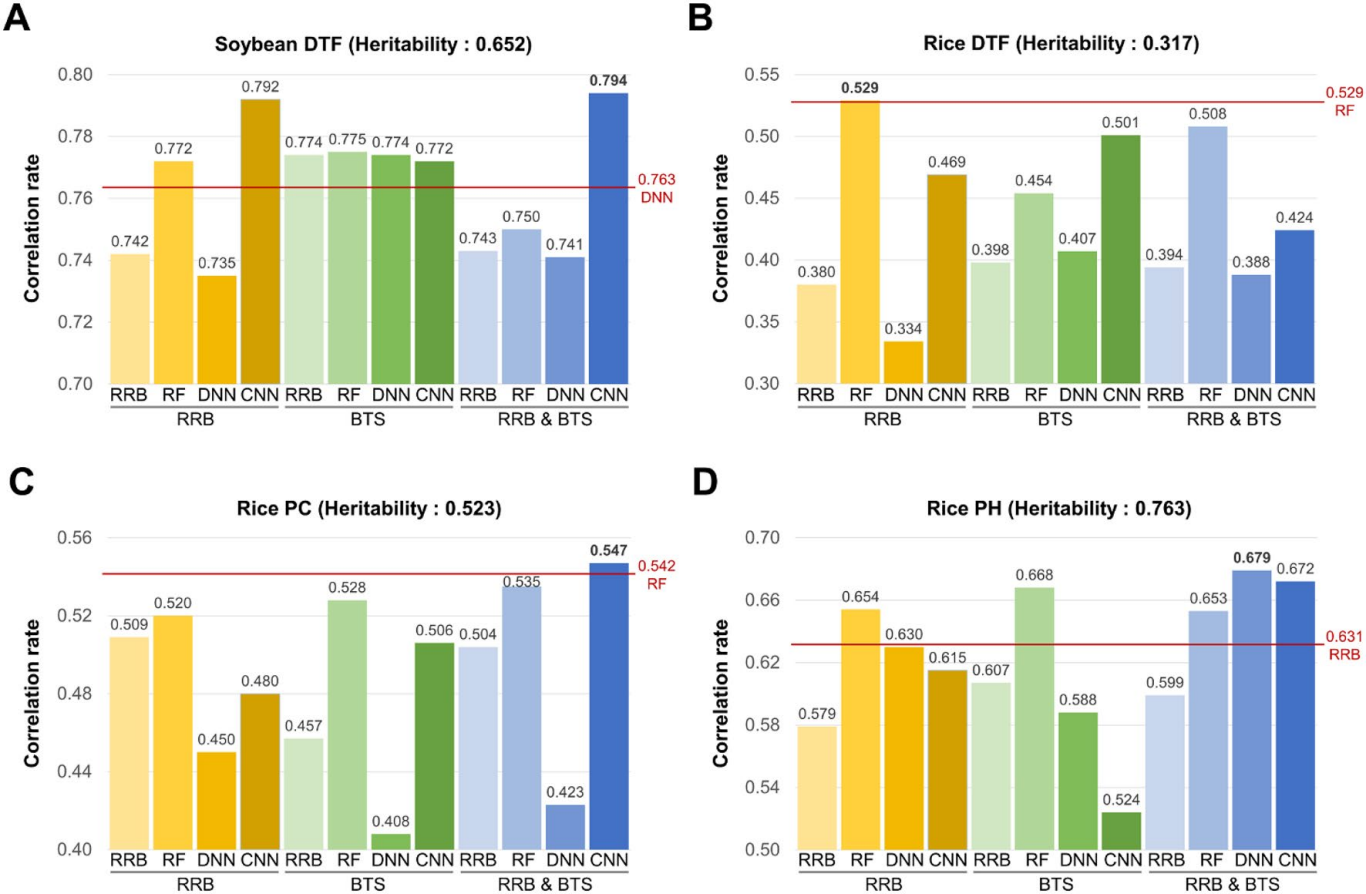
Correlation rates of test sets for four phenotypes, along with selection methods and prediction models. (**A**) Correlation rate of soybean DTF. (**B**–**D**) Correlation rates of rice DTF, PC, and PH. The *y*-axis indicates the correlation rate, and above and below the *x*-axis represents the selection methods and prediction models, respectively. The highest correlation rates for each phenotype are shown in bold, and the highest correlation rates when using all markers are indicated by a horizontal red line with that prediction model.

The modeling for GPs was conducted on all the marker sets selected by the three methods (Table 2), using all four prediction models, RRB, RF, DNN, and CNN (Fig. 4, Table 3). After the modeling, the test sets of the four phenotypes, which were not included during the marker selection phase, were used for phenotype prediction, and the correlation rates between these predicted phenotypes and their observed phenotypes were calculated as the metric of the accuracy for the selected marker sets. In rice, DTF had the highest correlation rate of 0.529, using the RRB selection method and the RF prediction model. PC and PH showed the highest correlation rates, 0.547 and 0.679, when selecting markers using RRB-BTS and predicting with CNN and DNN, respectively. Soybean-DTF had the highest correlation rate, 0.794, under the RRB-BTS selection method and CNN prediction model. Excluding rice-DTF, which had the lowest heritability, using the RRB-BTS selection method and the deep-learning model showed the best prediction performance. Generally, as heritability was higher, the correlation rate was also higher, and the predicted phenotypes exhibited stronger linearity with the observed phenotypes (Supplementary Fig. S2). In this result, the BTS selection method derives marker sets with the smallest number of markers, but its prediction performances were not significantly different compared to other selection methods. Under the best prediction models, the number of selected markers was 746, 873, 675, and 2256 in rice-DTF, PC, PH, and soybean-DTF, respectively (Table 3). These markers were distributed throughout the chromosomes, particularly in soybean DTF and PH, with high heritability. The number of markers for each chromosome was proportional to the GWAS-peak (Fig. 3 and Supplementary Fig. S3). Regarding computation time, RRB took the least time, followed by CNN, DNN, and RF (Supplementary Table S3). RRB and RF were computed using the Intel Xeon E5-2680 CPU, and DNN and CNN were computed using the Quadro RTX 6000 GPU. The larger the marker set, the more time tended to be required for completion.

**Table 3:** Final correlation rates of the selected marker sets on the test sets.

Data	Phenotype	Method	Selected markers	RRB (train/val./test)	RF (train/val./test)	DNN (train/val./test)	CNN (train/val./test)
Rice	DTF	RRB	746	0.997/0.870/0.380	0.976/0.845/**0.529**	0.932/0.912/0.334	0.948/0.895/0.469
		BTS	120	0.849/0.710/0.398	0.969/0.869/0.454	0.920/0.759/0.407	0.902/0.748/0.501
		RRB and BTS	817	0.996/0.854/0.394	0.977/0.847/0.508	0.977/0.895/0.388	0.942/0.840/0.424
	PC	RRB	805	0.998/0.831/0.509	0.974/0.742/0.520	0.873/0.811/0.450	0.917/0.833/0.480
		BTS	114	0.879/0.746/0.457	0.971/0.767/0.528	0.826/0.769/0.408	0.868/0.801/0.506
		RRB and BTS	873	0.998/0.845/0.504	0.975/0.745/0.535	0.907/0.802/0.423	0.937/0.854/**0.547**
	PH	RRB	620	0.992/0.924/0.579	0.981/0.915/0.654	0.977/0.932/0.630	0.956/0.909/0.615
		BTS	115	0.937/0.862/0.607	0.979/0.918/0.668	0.973/0.930/0.588	0.958/0.925/0.524
		RRB and BTS	675	0.992/0.923/0.599	0.981/0.918/0.653	0.922/0.905/**0.679**	0.943/0.903/0.672
SOY	DTF	RRB	2126	0.989/0.936/0.742	0.914/0.897/0.772	0.965/0.928/0.735	0.980/0.838/0.792
		BTS	224	0.853/0.821/0.774	0.816/0.820/0.775	0.855/0.839/0.774	0.973/0.823/0.772
		RRB and BTS	2256	0.989/0.935/0.743	0.922/0.907/0.750	0.967/0.923/0.741	0.980/0.837/**0.794**

Highest correlation rates in each phenotype are shown in bold.

### Performance comparison

To evaluate the performance of GMStool, additional tests were conducted on the whole marker sets, and the marker sets corresponding to the top 0.5%, 1%, 2%, and 3% of the significant GWAS hits (Table 4). These additional tests used the same test sets applied in the marker selection and final modeling. For the whole marker sets, the correlation rates of rice-DTF, PC, PH, and soybean-DTF were the highest at 0.529, 0.542, 0.631, and 0.763 in the RF, RF, RRB, and DNN prediction models, respectively. In rice-DTF with the lowest heritability of 0.317, the correlation rate of all markers was higher than those of GWAS-top markers and showed the same value as the correlation rate of the optimal marker set selected from GMStool (Fig. 4). For the other phenotypes with a heritability of > 0.5, the correlation rates of all markers were lower than those of the GWAS-top 3% markers as well as lower than those of the optimal marker sets. As heritability increases, the combinations of the selection-prediction methods with higher correlation rates than the whole marker set tended to increase. Among the GWAS-top marker sets, the top 3% marker sets showed the highest correlation rates (except rice-DTF), but all of them were lower than the GMStool’s optimal marker sets (Table 4). The optimal marker sets of PC and PH showed 1.003- and 1.015-times higher correlation rates with 21% and 39% fewer markers, respectively, and soybean-DTF showed 1.030 times higher correlation rate with 21% fewer markers than the GWAS-top 3% marker set. Overall, GMStool formed optimal marker sets with a relatively small number of markers and achieved better performance than the whole marker and GWAS-top 3% marker sets.

**Table 4:** Correlation rates of the GWAS-top 0.5%, 1%, 2%, and 3% marker-sets and whole marker-set.

Data	Phenotype	Selected markers^a^	RRB (train/val./test)	RF (train/val./test)	DNN (train/val./test)	CNN (train/val./test)
Rice	DTF	184	0.838/0.777/0.343	0.935/0.641/0.330	0.914/0.727/0.328	0.862/0.686/0.301
		369	0.890/0.709/0.480	0.963/0.570/0.464	0.945/0.717/0.412	0.905/0.656/0.410
		738	0.919/0.715/0.478	0.968/0.621/0.484	0.962/0.726/0.406	0.925/0.734/0.468
		1107	0.940/0.715/0.448	0.968/0.644/0.485	0.966/0.713/0.486	0.931/0.659/0.439
		All	0.844/0.412/0.515	0.964/0.398/**0.529**	0.899/0.366/0.512	0.923/0.363/0.504
	PC	184	0.816/0.744/0.366	0.947/0.500/0.376	0.851/0.664/0.304	0.834/0.680/0.308
		369	0.871/0.717/0.517	0.960/0.524/0.471	0.844/0.624/0.486	0.872/0.555/0.387
		738	0.917/0.761/0.508	0.963/0.513/0.521	0.874/0.619/0.532	0.891/0.708/0.402
		1107	0.944/0.760/**0.545**	0.968/0.495/0.537	0.958/0.776/0.531	0.912/0.732/0.422
		All	0.874/0.449/0.473	0.968/0.326/0.542	0.902/0.554/0.521	0.917/0.415/0.400
	PH	184	0.914/0.834/0.494	0.971/0.787/0.578	0.931/0.795/0.492	0.944/0.814/0.586
		369	0.946/0.859/0.571	0.976/0.808/0.617	0.934/0.823/0.579	0.950/0.772/0.643
		738	0.970/0.869/0.566	0.980/0.830/0.646	0.956/0.875/0.527	0.952/0.865/0.527
		1107	0.982/0.897/0.636	0.979/0.849/0.661	0.964/0.890/0.603	0.955/0.879/**0.669**
		All	0.985/0.733/0.631	0.972/0.737/0.548	0.912/0.765/0.624	0.972/0.737/0.548
SOY	DTF	478	0.641/0.558/0.578	0.744/0.644/0.627	0.663/0.660/0.628	0.566/0.578/0.584
		957	0.832/0.795/0.705	0.957/0.778/0.725	0.825/0.763/0.705	0.634/0.636/0.656
		1914	0.905/0.840/0.757	0.974/0.775/0.764	0.897/0.825/0.747	0.846/0.804/0.739
		2871	0.944/0.872/0.767	0.978/0.809/**0.771**	0.938/0.850/0.746	0.810/0.804/0.753
		All	0.973/0.743/0.753	0.976/0.752/0.760	0.921/0.751/0.763	0.904/0.721/0.748

Highest correlation rates in each phenotype are shown in bold.

^a^Rows of each phenotype represent the number of GWAS top 0.5%, 1%, 2%, and 3%, and all markers in order.

## Discussion

Selecting optimal markers reduces the dimensionality of genomic data for prediction and provides a small number of model parameters for better generalization in prediction modeling^13^. The prediction accuracy of an optimal marker set depends on how well it reflects the characteristics of the markers involved in a phenotype; thus, it is important to construct a marker-set with appropriate markers^14^. In this respect, many studies have adopted approaches that either directly exclude uninformative markers or assign weights to markers according to their contributions in a large set of markers^31–33^. These approaches have contributed to improving the accuracy of the GP, but simultaneously, it is difficult to select the appropriate markers to be excluded or the weight values to be assigned. In particular, when these approaches are based on GWAS, obtaining robust weights is problematic due to marker effects or *p*-values being calculated differently according to the GWAS methods^31^. Moreover, these approaches often require an amount of computation if they conduct modeling based on the large marker set. As a means of solving these problems, this study proposed a new approach tool, GMStool.

GMStool selects such optimal marker sets more effectively. Based on the prioritization of markers derived from a GWAS, GMStool sequentially searches for markers that increase the correlation rate between observed and predicted phenotypes and constructs an optimal marker set by accumulating these markers individually. As selection methods, the RRB model is provided to reflect the additive effects of markers on a phenotype, and the BTS model is provided to consider the interaction effects of markers, although indirect due to the use of GWAS. GMStool applies a *k*-fold CV approach with multi-threading and finally delivers an optimal marker set with minimal overfitting for a given dataset^34^. Additionally, to enable the flexible selection of optimal marker sets from various phenotypes, GMStool offers various options, such as a pre-selected marker list, initial markers, target correlation, increment rate of correlation, selection methods, and CV *k*-value (the –pre, –is, –c, –d, –m, and –cv options, respectively).

Evaluation of the optimal marker sets is essential for presenting them as predictive marker sets. No single model best predicts all phenotypes^35^; thus, to handle diverse phenotypes, GMStool provides four types of models in the prediction modeling: RRB, RF, DNN, and CNN. RRB assumes no interaction between markers but can predict phenotypes with high accuracy in practice^36^. RF has the ability to predict phenotypes by considering interaction effects as well as the dominance effects of markers^37^. DNN and CNN learn the global and local genotype patterns associated with a phenotype, thus can reflect the complicated relationship between genotype and phenotype without requiring predefined rules (e.g., normal distribution, non-zero equal variance)^24^. The DNN and CNN models of GMStool were built only to predict the optimal marker sets (Fig. 2) and are not provided in the marker selection phase because of the model complexity with various techniques applied to reduce overfitting (Fig. 2). Among the models provided, the RF, DNN, and CNN models can reflect the interaction effects of markers, but the interaction effects in the optimal marker set are considered restrictive since GMStool’s markers are selected based on the GWAS.

GMStool showed high performance on real datasets. The optimal marker sets of the rice-DTF, PC, PH, and soybean-DTF phenotypes were constructed as 746, 873, 675, and 2256 markers through RRB, RRB-BTS, RRB-BTS, and RRB-BTS selection methods, respectively (Table 3). The correlation rates of the test sets were 0.529, 0.547, 0.679, and 0.794, under RF, CNN, DNN, and CNN prediction models, respectively. In the selection methods, BTS selected the smallest number of markers, but their prediction performance was not far behind that of RRB (Table 3). Except for the rice-DTF with the lowest heritability, using both the RRB and BTS methods (RRB-BTS) showed better prediction performance than using either selection method. In particular, the RRB-BTS selection method showed the best performance when DNN or CNN was used as a prediction model. Using a phenotype with a heritability of > 0.5, it is recommended to use the combination of the RRB-BTS selection method and the deep-learning prediction model that can consider the complicated relationship between markers and phenotype. In PC, PH, and soybean-DTF with a heritability of > 0.5, GMStool's optimal marker sets showed higher prediction performance than those using whole marker sets (Fig. 4). In rice-DTF with a lower heritability of 0.317, the prediction performance was the same for the optimal marker set and the whole marker. GMStool's optimal marker sets had a much smaller number of markers but showed at least the same performance as those using all markers. However, in the case of using a phenotype with very low heritability, using all markers for prediction is recommended. This is because a large number of markers have a very low genetic effect, so a subset of all the markers has a fundamental limitation in improving the prediction performance. In comparison with GWAS-top markers, GMStool’s optimal marker sets had an average of 27% fewer markers than the GWAS-top 3% marker sets and achieved a 1.004- to 1.088-times higher performance than these marker sets (Table 4). Overall, the GMStool constructed the optimal marker set well and showed relatively high prediction performance in the real datasets evaluated. Although GMStool has a simple algorithm, it is expected to achieve high performance on other real datasets.

Nevertheless, GMStool has several limitations. First, marker selection is influenced by the GWAS results. GMStool uses the priority of the markers derived from the GWAS result and sequentially selects markers with the lowest *p*-value. This approach has less dependence on the GWAS result than other tools that directly use the marker effects or *p*-values, but it can also derive an inappropriate marker set with low prediction accuracy if the GWAS is incorrect. One thing to be aware of when using GWAS results is that GWAS must be performed without the test set. Otherwise, an overfitted marker set may result from the reflected information for the test set^16^. In addition, since high levels of linkage disequilibrium (LD) between SNPs can affect prediction performance, it is recommended to perform SNP pruning for LD before GWAS or clumping after GWAS. Second, the interaction effects of markers on the phenotype considered are indirect. Since GWAS only considers the linear effect of a single marker, GMStool based on this result has limited consideration of markers' interaction effect in RF, BTS, CNN, and DNN models. Despite these constraints, the models indirectly considering the interaction effects had occasions that showed better performances than the models that considered only linear effects in the evaluated data set. Third, the results of GMStool are affected by the input options, such as initial markers, *k*-CV value, increment value, and target correlation rate. Since these options are provided so that the user can flexibly cope with various phenotypes, the user should preferentially find optimal options for the target phenotype through several pretests combining these options. As for the number of initial markers, the top one GWAS marker was used as the default option under the assumption that the GWAS result is reasonable. If the GWAS result is highly reliable, the user may designate the initial markers to the markers related to phenotype from the top GWAS markers; otherwise, it is recommended to directly designate the related markers as initial markers the ‘pre-selected marker’ option. The *k-*value of CV is suggested to be between 3 and 5 depending on the training sample size. The increment value for the correlation rate is recommended to be at least 0.00005 to avoid excessive selection of SNPs within the same LD block, and this setting is particularly recommended if pre-processing or post-processing for LD is not performed. The target correlation rate is suggested to be at least 0.99 so that all potential markers can be selected, although the heritability of the phenotype to be used is low. In the case of low heritability, even if all potential markers for the train and validation set are selected, the final prediction performance for the test set may not be superior to the prediction performance using all markers (Fig. 4B). Despite these limitations, GMStool is expected to contribute to many studies predicting various quantitative phenotypes with genotypes.

## Supplementary information

Supplementary Information 1.

Supplementary Information 2.

Supplementary Information 3.

## Data Availability

The program GMStool developed in this study is freely available at www.github.com/JaeYoonKim72/GMStool, with detailed usage instructions and example files.
