# Supplementary material for: GMStool: GWAS-based marker selection tool for genomic prediction from genomic data
Source: Sci Rep. 2020 Nov 12;10:19653. doi: 10.1038/s41598-020-76759-y (PMC7665227; doi:10.1038/s41598-020-76759-y)

Supplementary Information 3

GMStool: GWAS-based Marker Selection Tool for Genomic Prediction from Genomic Data

Seongmun Jeong^1,†^, Jae-Yoon Kim^1,2,†^, and Namshin Kim^1,2,*^

^1^Genome Editing Research Center, Korea Research Institute of Bioscience and Biotechnology, Daejeon 34141, Republic of Korea

^2^Department of Bioinformatics, KRIBB School of Bioscience, University of Science and Technology (UST), Daejeon 34141, Republic of Korea.

†These authors contributed equally to this research

^*^Corresponding author

**Contents**

Tables S1-S3

Figures S1-S3

*Tables S1-S2 are separately provided as excel files, named “Supplementary information 1 and 2".

**Supplementary table S3.** Run time for marker selection and final modeling

| Data | Phenotype | Method | Marker selection | | | Final modeling | | | | |
| --- | --- | --- | --- | --- | --- | --- | --- | --- | --- | --- |
|  |  |  | CV | Time^a^ | Markers | | RRB | RF | DNN | CNN |
| Rice | DTF | RRB | 3 | 4h 52m | 746 | | <1m | 31m | 26m | 16m |
|  |  | BTS | 3 | 4h 25m | 120 | | <1m | 20m | 14m | 11m |
|  |  | RRB&BTS | 3 | 9h 24m | 817 | | <1m | 32m | 27m | 15m |
|  | PC | RRB | 3 | 5h 52m | 805 | | <1m | 31m | 24m | 19m |
|  |  | BTS | 3 | 4h 48m | 114 | | <1m | 20m | 19m | 15m |
|  |  | RRB&BTS | 3 | 10h 47m | 873 | | <1m | 38m | 29m | 23m |
|  | PH | RRB | 3 | 6h 46m | 620 | | <1m | 31m | 22m | 19m |
|  |  | BTS | 3 | 6h 3m | 115 | | <1m | 19m | 18m | 13m |
|  |  | RRB&BTS | 3 | 12h 55m | 675 | | <1m | 31m | 23m | 21m |
| SOY | DTF | RRB | 5 | 15h 6m | 2,126 | | 2m | 2h 10m | 34m | 27m |
|  |  | BTS | 5 | 8h 2m | 282 | | <1m | 1h 1m | 26m | 25m |
|  |  | RRB&BTS | 5 | 23h 32m | 2,256 | | 2m | 2h 29m | 33m | 29m |

^a^Average run time per CV, applied with the option to stop when the correlation rates of the validation set do not improve continuously by a number equal to 20% of the total number of input markers.

**Supplementary figure S1.** Distribution of the training and test samples on principal component analyses. (A) Plot of soybean DTF. Principal components 1 and 2 account for 74.51% and 10.64% of the total genotypic variance, respectively. (B-D) Plots of rice DTF, PC, and PH. Principal components 1 and 2 of the DTF explain 95.01% and 2.52% of the total genotypic variance, and those of PC and PH explain 94.97% and 2.72%, and 94.85% and 2.78% of their total genotypic variance, respectively.


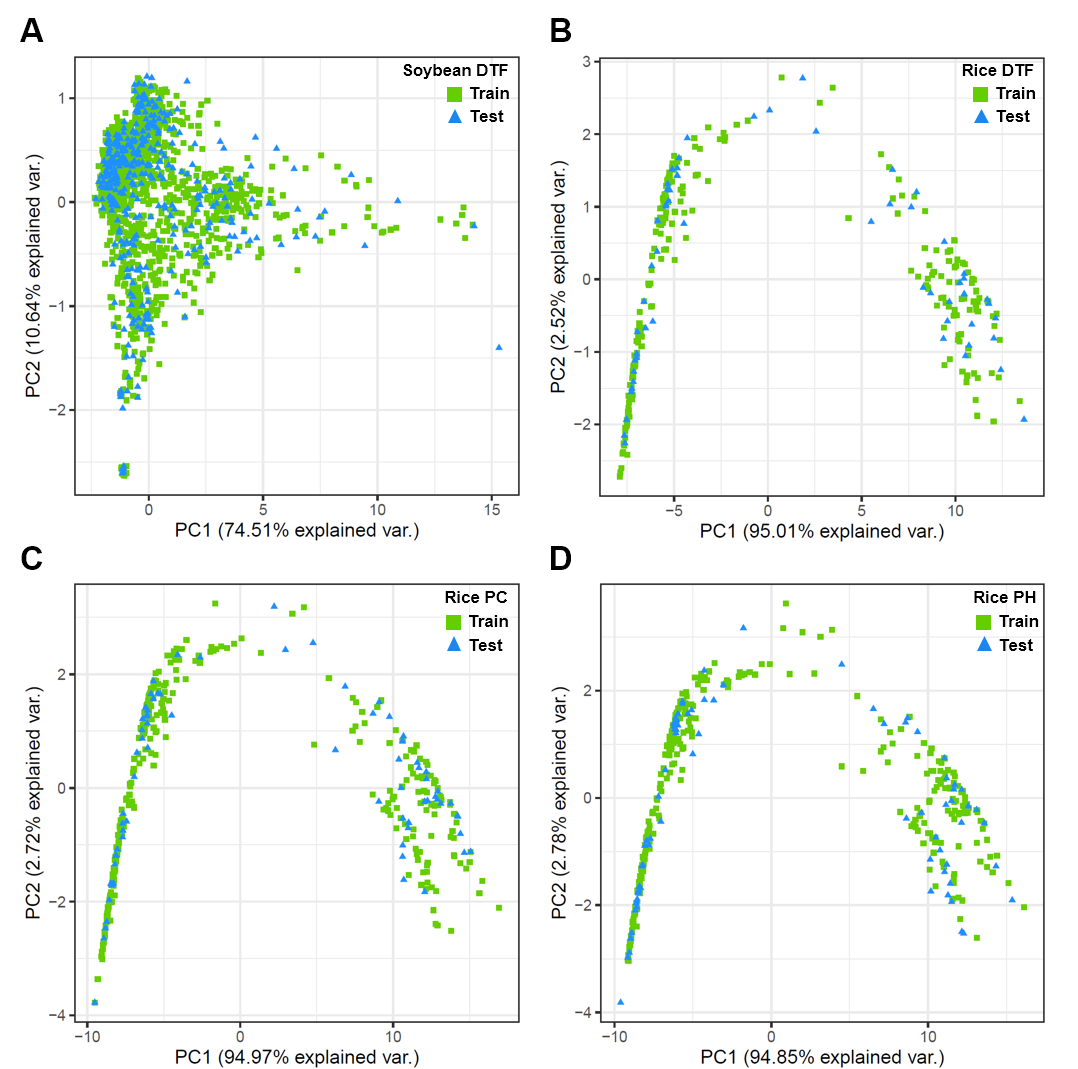


**Supplementary figure S2.** Predicted and observed phenotype values in the best prediction model. (A) Scatter plot of soybean DTF under the CNN prediction model. (B-D) Scatter plots of rice DTF, PC, and PH, where the RF, CNN, and DNN models were used as prediction models, respectively. Linear model equations are shown at the top-left of the plots along with their R-squared values.


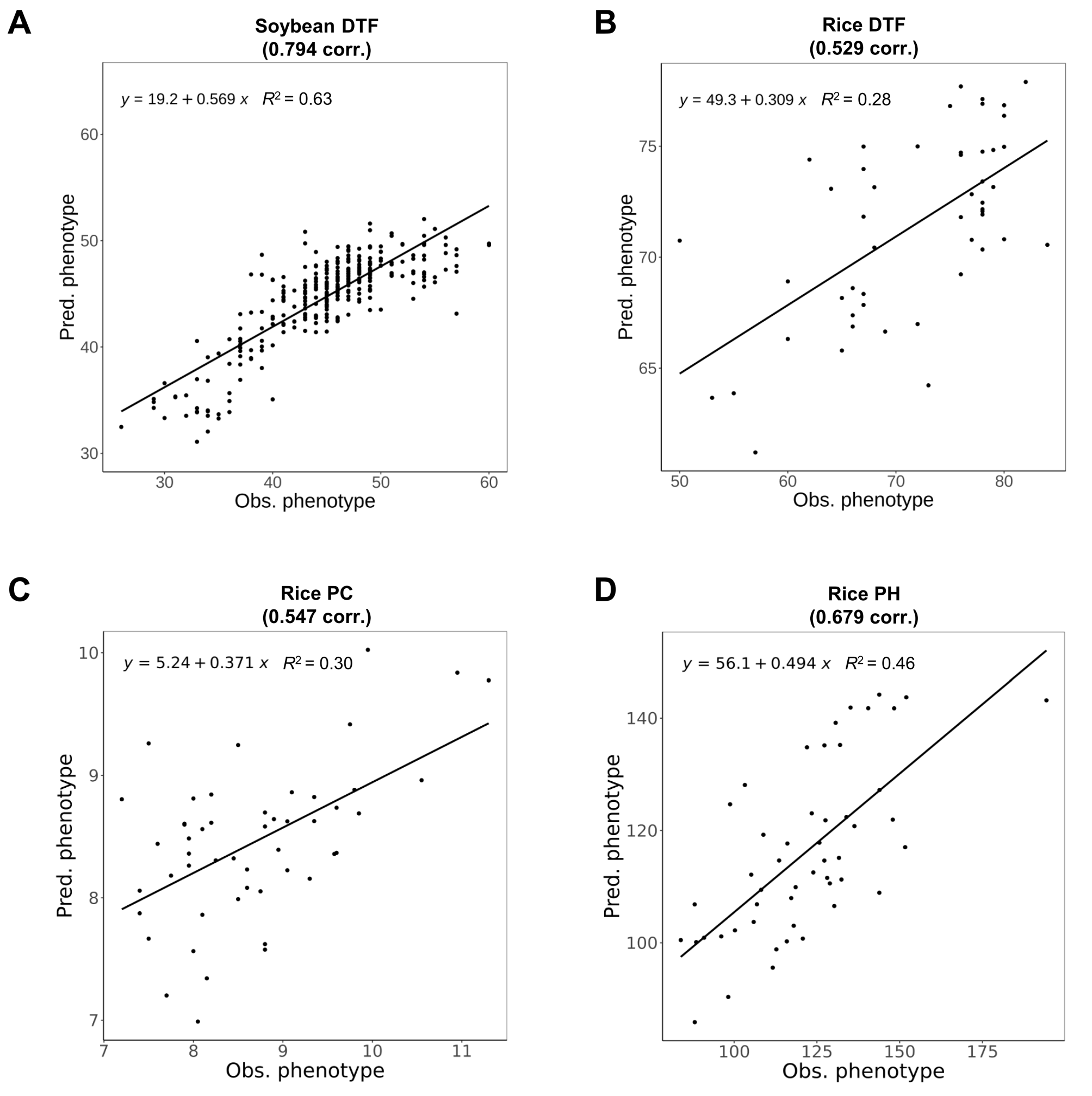


**Supplementary figure S3.** Chromosomal distribution of selected markers in the best prediction model. (A) Bar plot of soybean DTF markers selected by using both RRB and BTS methods. (B-D) Bar plots of rice DTF, PC, and PH markers were selected using RRB, RRB-BTS, and RRB-BTS methods, respectively. The total number of selected markers is indicated at the top of the plot, and the number of markers in each chromosome is indicated on each bar in the plot.


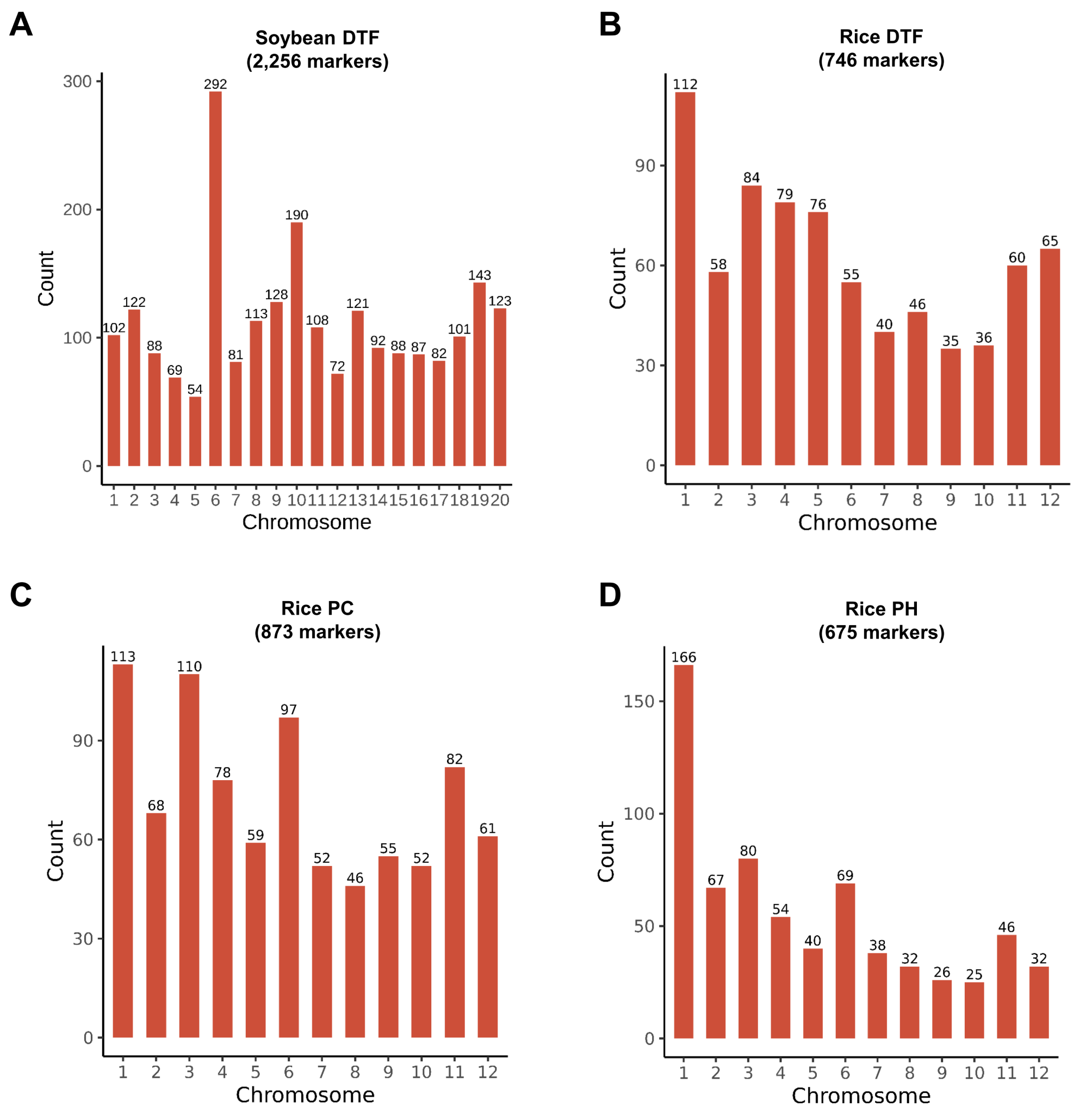

Supplement: Supplementary file 1 — Supplementary Information 1. [file 41598_2020_76759_MOESM1_ESM.docx]
